# A Robust Semi-Supervised Brain Tumor MRI Classification Network for Data-Constrained Clinical Environments

**DOI:** 10.3390/diagnostics15192485

**Published:** 2025-09-28

**Authors:** Subhash Chand Gupta, Vandana Bhattacharjee, Shripal Vijayvargiya, Partha Sarathi Bishnu, Raushan Oraon, Rajendra Majhi

**Affiliations:** Department of Computer Science and Engineering, BIT, Mesra, Ranchi 835215, India; sgupta@bitmesra.ac.in (S.C.G.); svijayvargiya@bitmesra.ac.in (S.V.); psbishnu@bitmesra.ac.in (P.S.B.); btech10374.22@bitmesra.ac.in (R.O.); btech10332.22@bitmesra.ac.in (R.M.)

**Keywords:** semi-supervised learning, pseudo-labelling, brain tumor classification, Magnetic Resonance Imaging (MRI), confidence thresholding

## Abstract

**Background:** The accurate classification of brain tumor subtypes from MRI scans is critical for timely diagnosis, yet the manual annotation of large datasets remains prohibitively labor-intensive. **Method:** We present SSPLNet (Semi-Supervised Pseudo-Labeling Network), a dual-branch deep learning framework that synergizes confidence-guided iterative pseudo-labelling with deep feature fusion to enable robust MRI-based tumor classification in data-constrained clinical environments. SSPLNet integrates a custom convolutional neural network (CNN) and a pretrained ResNet50 model, trained semi-supervised using adaptive confidence thresholds (τ = 0.98 → 0.95 → 0.90) to iteratively refine pseudo-labels for unlabelled MRI scans. Feature representations from both branches are fused via a dense network, combining localized texture patterns with hierarchical deep features. **Results:** SSPLNet achieves state-of-the-art accuracy across labelled–unlabelled data splits (90:10 to 10:90), outperforming supervised baselines in extreme low-label regimes (10:90) by up to 5.34% from Custom CNN and 5.58% from ResNet50. The framework reduces annotation dependence and with 40% unlabeled data maintains 98.17% diagnostic accuracy, demonstrating its viability for scalable deployment in resource-limited healthcare settings. **Conclusions:** Statistical Evaluation and Robustness Analysis of SSPLNet Performance confirms that SSPLNet’s lower error rate is not due to chance. The bootstrap results also confirm that SSPLNet’s reported accuracy falls well within the 95% CI of the sampling distribution.

## 1. Introduction

Brain tumors describe one of the most critical and life-threatening forms of cancer, with an early and accurate diagnosis being vital for effective treatment planning and prognosis. Magnetic Resonance Imaging (MRI) is a widely adopted, non-invasive imaging modality that supplies a detailed visualization of brain structures and anomalies, enabling the detection of tumors such as glioma, meningioma, and pituitary adenomas. The automatic classification of brain tumor types from MRI images using deep learning (DL) [1,2,3,4,5,6,7,8,9,10] and machine learning has shown significant promise in aiding clinical decision-making and reducing radiologist workload.

Despite the remarkable performance of deep learning models in medical image classification, their success heavily relies on large volumes of labelled data. Annotating MRI scans needs domain expertise, is time-consuming, and often suffers from inter-observer variability. This limitation creates a bottleneck in training robust models, especially in real-world settings where annotated data is scarce, but unlabelled scans are abundant. Existing supervised learning approaches struggle in such low-label regimes, leading to a reduced generalizability and model performance. The purpose of this paper is to study the role of semi-supervised learning in the improvement of a classification model for low-label regimes.

**Figure 1:**
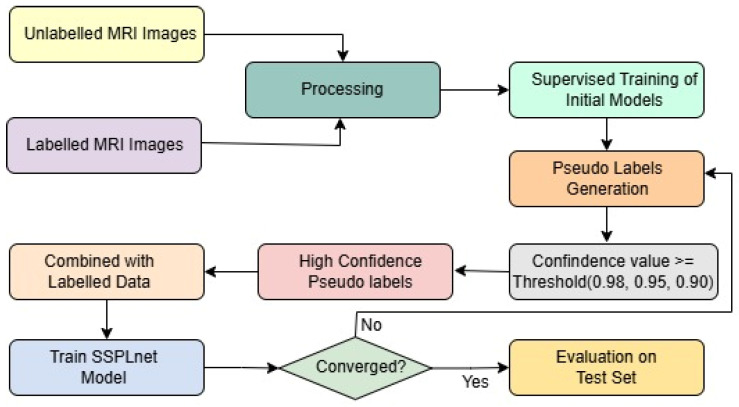
Framework of the proposed work, including pseudo-labeling and SSPLNet.

To address this challenge, we introduce the Semi-Supervised Pseudo-Labeling Network (SSPLNet), a robust semi-supervised framework for brain tumor MRI classification that integrates confidence-guided iterative pseudo-labelling with dual-branch deep feature fusion. SSPLNet leverages a limited set of labelled MRI images alongside a large repository of unlabelled scans by generating high-confidence pseudo-labels through its dual-branch architecture. These pseudo-labelled samples are iteratively incorporated into the training process, with adaptive confidence thresholds (e.g., τ = 0.98, 0.95, 0.90) progressively guiding the selection and refinement of labels. This approach not only enhances model accuracy by effectively utilizing unlabelled data but also significantly reduces the dependence on manual annotation. The comprehensive workflow of the proposed method, including the fusion of features from both branches, is illustrated in Figure 1.

The key contributions of the paper are as follows:

- Labelled data is utilized to train a custom convolutional network model and a pretrained ResNet50 model as baseline supervised classifiers.
- A confidence-based iterative pseudo-labeling strategy is applied to unlabelled data, and semi-supervised training of SSPLNet is performed.
- For the final classification, a dense layer network is designed for feature fusion.
- A comprehensive comparative study is conducted by varying key parameters to evaluate model performance across different labelled–unlabelled distributions.
- To evaluate the improvement offered by the proposed semi-supervised approach, we conducted a paired *t*-test comparing the accuracy values of supervised (baseline) and semi-supervised learning across all labelled–unlabelled splits.

The rest of the paper is organized as follows: Section 2 presents the related work on brain tumor classification and semi-supervised learning approaches. Section 3 describes the methodology of SSPLNet, including pseudo-label generation, confidence-based filtering, and the iterative training procedure. Section 4 discusses the experimental setup, including dataset details, parameter settings, and evaluation metrics. Section 5 presents the results and analysis, beginning with the pseudo-labeling strategy using a custom CNN, followed by the performance of the SSPLNet dual-branch model. An ablation study is then conducted to assess the impact of feature fusion versus individual model performance. A comparison with existing state-of-the-art models is also provided. Section 6 comprehensively discusses the findings, implications, and limitations. Finally, Section 7 concludes the paper and outlines potential directions for future research.

## 2. Related Work

In this section, we review recent research on brain tumor classification using deep learning and semi-supervised approaches. In [1], a convolutional neural network (CNN)-based deep learning model is proposed to classify various brain tumor types using two publicly available datasets. The authors of [2] present a two-phase deep learning framework for tumor detection, while studies [3,4,5] explore ensemble and hybrid methods to address the same challenge. ZainEldin et al. [6] employ Grey Wolf Optimization for brain tumor detection. Additionally, several researchers [7,8,9,10] have applied CNN-based techniques to tackle this problem.

Semi-supervised learning, or SSL, has become a popular way to use large amounts of unlabelled data, and is especially helpful in domains like medical imaging, where labelling data is expensive and requires expert knowledge. Oliver et al. [11] raised concerns about the evaluation framework for SSL methods and emphasized the need for consistent baselines to assess the benefits of unlabelled data properly. Their work highlights the many performance gaps and challenges between SSL and fully supervised models.

Chapelle et al. [12,13] explored algorithms such as semi-supervised SVMs and manifold regularization. These approaches have highlighted the potential of SSL in tasks where labelled data is limited, but were often restricted by the scalability and complexity of model tuning. Later, Triguero et al. [14] conducted a detailed analysis of self-labelling techniques, including pseudo-labelling methods, showing how such methods can be effective across various domains, including biomedical applications.

Deep learning models have further amplified and reshaped how representation learning is handled in supervised and semi-supervised contexts. The introduction of ResNet by He et al. [15] significantly improved model depth and stability, which is especially useful for complex image classification tasks such as brain tumor diagnosis. Techniques for the visual interpretability and understanding of CNNs were presented by Zeiler and Fergus [16]. At the same time, foundational challenges in deep learning were addressed by Bengio et al. [17] and Glorot and Bengio [18], who explored issues in gradient propagation and activation functions. This paper [19] introduces the Parametric ReLU (PReLU) activation and a robust initialization method, enabling deeper networks to be trained effectively. Their models surpassed human-level accuracy on the ImageNet classification benchmark. The authors [20] explore design strategies for CNNs under computational constraints, balancing accuracy and efficiency. They demonstrate that careful architecture choices can significantly reduce inference time without sacrificing performance.

In the context of brain MRI classification, representation learning methods have evolved through sparse autoencoders [21], denoising autoencoders [22], and GAN-based architectures [23], facilitating unsupervised and semi-supervised learning. 

These approaches allow neural networks to learn meaningful features even with unlabelled data, which gives a crucial advantage in medical domains. SSCLNet by Mishra et al. [24] proposed a self-supervised contrastive learning framework for brain MRI classification, which aligns well with pseudo-labelling techniques for leveraging unlabelled data. Moreover, several studies have shown the success of self-supervised learning in domains such as human activity recognition [25], histopathological image analysis [26,27,28,29,30], and brain tumor classification. This further demonstrates the adaptability and effectiveness of unsupervised and semi-supervised techniques across diverse biomedical image modalities. Researchers in [31] have proposed a semi-supervised approach, SSBTCNet, in which they combine an unsupervised Autoencoder (AE) with supervised classification networks. Authors in [32] apply MobileNet architecture for brain tumor classification. In [33], five popular deep learning architectures are utilized to develop a system for diagnosing brain tumors. The architectures used in this paper are XceptionNet, DenseNet201, DenseNet121, ResNet152V2, and InceptionResNetV2. As more and more researchers [34,35] delve into the field of brain tumor classification, it is becoming an appealing topic for exploring paradigms such as semi-supervised learning, hence the motivation for the present work. Authors in [36] use the features fusion technique to improve the accuracy of the model. The paper [37] surveys deep semi-supervised learning methods for medical image segmentation, focusing on how limited labelled data can be effectively leveraged.

The paper [38] proposes a unified taxonomy, highlights advancements, and emphasizes the role of unlabelled data in improving generalization. Lee and Cho [39] proposed a semi-supervised image classification method that leverages Grad-CAM consistency to improve model interpretability and performance. Gupta and Goel in paper [40] developed predictive models for diabetes diagnosis using machine learning techniques with hyperparameter tuning to improve accuracy. Chang et al. [41] applied various machine learning algorithms to classify diabetes in the Pima Indians dataset. Tigga et al. [42] explored multiple machine learning classification methods to predict type 2 diabetes using clinical data.

These studies collectively form the design of our work, which explores iterative pseudo-labelling with progressively relaxed confidence thresholds. This strategy allows us to incorporate unlabelled MRI images, resulting in consistent performance improvements, particularly when labelled data is scarce.

## 3. Methodology

This section outlines the proposed semi-supervised learning framework based on confidence-guided iterative pseudo-labelling for brain tumor classification using MRI images. The methodology involves preprocessing, supervised training, pseudo-label generation, sample filtering, and iterative retraining.

Let us denote the following: $D_{l}={\left\{(x_{i},y_{i})\right\}}_{i=1}^{N_{l}}$: the labelled dataset with $N_{l}$ samples, where $x_{i}\in R^{d}$ and $y_{i}\in \{1,2,\ldots ,C\}$. $D_{u}={\left\{(x_{j})\right\}}_{j=1}^{N_{u}}$: the unlabelled dataset with $N_{u}$ samples. $f_{\theta }(\cdot )$: the neural network with parameters θ. ${\hat{y}}_{j}$ = argmax $f_{\theta }(x_{j})$: the pseudo-label assigned to unlabelled input $x_{j}$. τ: confidence threshold for pseudo-label selection. $l_{sup}$: supervised loss (cross-entropy). $l_{unsup}$: unsupervised pseudo-label loss. λ: weighting coefficient for the unsupervised loss.

1. *Supervised Loss*: For labelled data, the standard cross-entropy loss is applied:
  (1) $$L_{sup}=\frac{1}{N_{l}}\sum_{i=1}^{N_{l}}l_{CE}(f_{\theta }\left(x_{i}\right),y_{i})$$
  where $l_{CE}$ is the categorical cross-entropy loss function.
2. *Confidence-Guided Pseudo-Labelling*: For unlabelled data $x_{j}\in D_{u}$, pseudo-labels are generated only if the model’s softmax confidence exceeds a threshold τ
  (2) $${\hat{y}}_{j}=\mathrm{argmax}\;f_{\theta }(x_{j})\;\mathrm{if}\;max\;f_{\theta }(x_{j})\geq \;\tau$$
  The filtered set of pseudo-labelled samples are given as follows:
  (3) $${\tilde{D}}_{u}=\{(x_{j},{\hat{y}}_{j})|max\;f_{\theta }(x_{j})\geq \tau \}$$
3. *Unsupervised Pseudo-Label Loss*: The pseudo-labels are treated as ground truth with cross-entropy loss:
  (4) $$L_{unsup}=\;\frac{1}{\left|{\tilde{D}}_{u}\right|}\sum_{\left(x_{j},\;{\hat{y}}_{j}\right)\in {\tilde{D}}_{u}}l_{CE}(f_{\theta }\left(x_{J}\right),{\hat{y}}_{j})$$
4. *Total Loss Function*: The total training objective combines the supervised and unsupervised components:
  (5) $$L_{total}=L_{sup}+\lambda \;*\;L_{unsup}$$
  where λ balances the contribution of pseudo-labelled data. It can be ramped up over training epochs.
5. *Iterative Rejection Strategy*: An iterative filtering mechanism is applied:
  - At each iteration t, pseudo-labels are re-evaluated based on confidence.
  - Rejected samples (with low confidence) are excluded in future iterations.
  - The process repeats until convergence or max iterations T.

In other words, the following is true:

(6) $${\tilde{D}}_{u}^{\left(t\right)}\;=\;\{(x_{j},\;{\hat{y}}_{j})\;|\;max\;f_{\theta }^{\left(t\right)}(x_{j})\geq \;\tau \},\;t\leq T$$

For the sake of completeness, the algorithmic steps of the proposed SSPLNet are outlined below in Algorithm 1.
**Algorithm 1:** SSPLNet—Semi-Supervised Pseudo-Labelling Network***Input***: Labelled dataset $D_{l}\;=\;{\left\{(x_{i},\;y_{i})\right\}}_{i=1}^{N_{l}}$, Unlabelled dataset. $D_{u}\;=\;{\left\{(x_{j})\right\}}_{j=1}^{N_{u}}$, Neural network model $f_{\theta }$, Confidence threshold τ∈[0,1], Loss weights: λ for pseudo-label loss, Max iterations T for pseudo-label refinement ***Output***: Trained model $f_{\theta }$ with updated parameters, Final labelled set including selected pseudo-labelled samples
Initialize model parameters θ randomly or from pre-trained weights.For each epoch (until convergence):2.1Train on labelled data: $\theta \leftarrow \theta -\eta \nabla_{\theta }\theta L_{sup}$, where $L_{sup}=\frac{1}{N_{l}}\sum_{i=1}^{N_{l}}l_{CE}(f_{\theta }\left(x_{i}\right),y_{i})$2.2Generate pseudo-labels for all$\;x_{j}\in D_{u}$:2.2.1If ${max}_{k}f_{\theta }(x_{j})\geq \;\tau$, assign: ${\hat{y}}_{j}$ = $\arg\underset{k}{max}{f_{\theta }(x_{j})}_{k}$2.2.2Store as pseudo-labelled data ${\tilde{D}}_{u}$2.3Compute unsupervised loss: $L_{unsup}=\;\frac{1}{\left|{\tilde{D}}_{u}\right|}\sum_{\left(x_{j},\;{\hat{y}}_{j}\right)\in {\tilde{D}}_{u}}l_{CE}(f_{\theta }\left(x_{J}\right),{\hat{y}}_{j})$2.4Update model using joint loss: $L_{total}\;=\;L_{sup}\;+\;\lambda \;*\;L_{unsup}$2.5Optional Iterative Rejection (if pseudo-labels are unstable):
▪Remove pseudo-labelled samples with confidence <τ▪Repeat pseudo-labelling up to TReturn trained model $f_{\theta }$


The time complexity of the algorithm is as follows: Supervised training: $O(E\cdot N_{l}\cdot C\cdot d),$ Pseudo-label generation: $O(T\cdot N_{u}\cdot C\cdot d)$, Overall complexity: $O(E\cdot N_{l}\cdot C\cdot d)$ + $O(T\cdot N_{u}\cdot C\cdot d)$, where E: number of epochs, T: max pseudo-label iterations, d: input dimensionality, C: number of classes.

## 4. Experiments

To evaluate the effectiveness of the proposed semi-supervised framework, a series of experiments were conducted using a publicly available brain tumor MRI dataset.

### 4.1. Dataset

**Figure 2:**
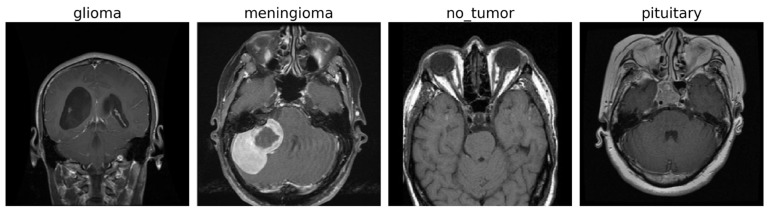
MRI images of brain tumor and normal brain.

The experiments were conducted on a publicly available brain tumor MRI dataset [43] consisting of a total of 7022 T1-weighted contrast-enhanced MRI images of human brain scans. These images are categorized into four distinct classes: glioma (G), meningioma (M), pituitary tumor (P), and no tumor (NT). Each image is a grayscale scan, captured in axial view, and provides high-resolution structural information suitable for tumor identification and classification. Figure 2 shows some images for the tumor classes and no tumor:

Out of the entire dataset, 5712 samples were used for training purposes, while the remaining 1311 samples were reserved as a fixed test set for evaluating model performance across all experimental configurations. The labelled-to-unlabelled data ratio varied systematically in the training set, ranging from 90:10 down to 10:90, to assess the robustness of the semi-supervised learning framework under different supervision levels. Each subset was stratified to maintain class balance and prevent distributional bias across training and evaluation phases.

### 4.2. Parameter Settings

The custom CNN model comprises four convolutional blocks, each followed by max-pooling, and finally flattening with a dense layer of 512 neurons and output layer of 4 neurons. It was trained using the Adam optimizer with an initial learning rate of α = 0.001, and momentum parameters β1 = 0.85 and β2 = 0.9925. To dynamically adjust the learning rate during training, two scheduling mechanisms were employed. The first, a custom callback named “Reduce LR On Multiple Accuracies,” reduced the learning rate by a factor of 0.75 whenever the validation accuracy surpassed certain thresholds, specifically at 0.96, 0.99, and 0.9935. A minimum learning rate of $1\;\times \;10^{-4}$ was enforced to prevent excessive decay. This dual-learning rate adaptation strategy helped maintain learning momentum while avoiding overshooting once the model approached convergence.

The training was conducted with a batch size of 32 for 50 epochs. To ensure consistent model input and enhance generalization, a comprehensive preprocessing pipeline was designed and implemented. The pipeline incorporated dataset organization, image decoding and transformation, data augmentation, and normalization. The input MRI images were first resized to a fixed shape, followed by data augmentation that included random horizontal flipping, small-angle rotations (up to 2%), contrast adjustments (within a 10% range), zooming, and spatial translations. All pixel values were normalized to the [0, 1] range by dividing by 255. This operation was applied uniformly across both the labelled and unlabelled datasets. For the training set, normalization followed augmentation to maintain the integrity of the applied transformations. For the test and validation datasets, only normalization was applied, ensuring consistency and unbiased performance evaluation.

### 4.3. Evaluation Parameters

Model performance was evaluated using a separate test set consisting of 1311 MRI images, which remained fixed across all experimental configurations. The primary evaluation metric was classification accuracy, which quantified the proportion of correctly predicted labels across all test samples. Other metrics like precision, recall, and F1-score were also computed. Experiments were conducted across nine labelled-to-unlabelled data splits ranging from 90:10 to 10:90.

During each pseudo-labelling iteration, only those unlabelled samples with model confidence exceeding a predefined confidence threshold (τ∈{0.98,0.95,0.90}) were selected for augmentation of the labelled training set. The confidence thresholds τ = (0.98, 0.95, 0.90) were selected to balance the trade-off between label reliability and data utilization in the pseudo-labelling process. The higher thresholds (e.g., 0.98) ensure that only highly confident predictions are incorporated initially, minimizing the risk of propagating incorrect labels. As training progresses, the thresholds are gradually lowered (0.95, 0.90) to allow the model to leverage a larger portion of unlabelled data while maintaining sufficient accuracy. This adaptive strategy is consistent with common practices in semi-supervised learning, where progressively relaxing the confidence criteria helps the model to refine pseudo-labels iteratively and improve generalization without heavily relying on manual annotations. The performance of the semi-supervised approach was benchmarked against purely supervised training under the same configurations to evaluate improvements in classification outcomes.

### 4.4. SSPLNet: Fusing ResNet50 and Custom CNN

In this section, we present the details of our proposed SSPLNet model, elaborating the input modalities, the architectural details of the two models, and finally the feature fusion and classification head.

#### 4.4.1. Input Modalities

For the ResNet50 [15] branch, the input is an RGB image of size 224 × 224 × 3, and for the custom CNN branch the input is an image resized to 168 × 168 × 1. Each image is preprocessed accordingly, normalized, and then passed through its respective feature extractor. A custom data pipeline is built to simultaneously supply both RGB and grayscale versions of the same image to the respective model branches. Each image is loaded once, preprocessed in two formats, and passed as a dual-input tuple to the model. Labels are encoded as one-hot vectors for compatibility. For the categorical cross-entropy loss function, an Adam optimizer with a learning rate of 0.0001 is used for model compilation.

#### 4.4.2. Architecture Details

We now discuss the pseudo-labelling framework with dual network architecture and deep hybrid model design integrating ResNet50 [15] and custom CNN.

##### Pseudo-Labelling Framework with Dual Network Architecture

**Figure 3:**
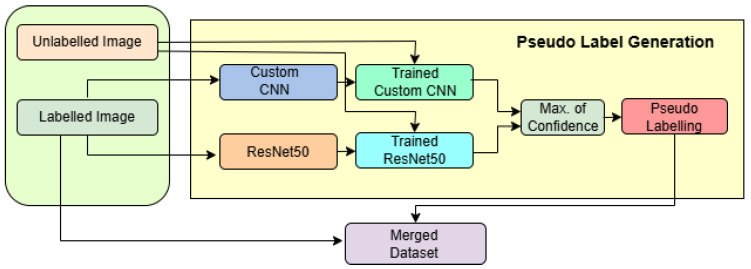
Workflow of the pseudo-label generation framework using custom CNN and ResNet50.

In Figure 3, the semi-supervised pseudo-labelling framework is depicted, showcasing a dual-model architecture that combines the strengths of a pretrained ResNet50 and a custom-designed convolutional neural network (CNN) to enhance classification performance on brain MRI images. The framework is designed to handle both labelled and unlabelled data in a robust and structured pipeline.

The process begins with two input streams: labelled MRI images and unlabelled images. The labelled images are used to independently train two models. The first branch utilizes a ResNet50 backbone pretrained on ImageNet, configured with an input shape of 224 × 224 × 3. The top layers are excluded, and feature extraction is followed by a GlobalAveragePooling2D layer with Dropout(0.5), a dense layer with 128 neurons and ReLU activation, another Dropout(0.5), and finally a softmax layer with 4 output units. The model is compiled with the Adamax optimizer (with learning rate = 0.0001) and trained using a model checkpoint callback for saving the best model based on validation accuracy. The data is loaded using ImageDataGenerator with pixel normalization (rescale = 1/255) and is provided in mini-batches of size 32.

In parallel, the second branch uses a custom CNN designed for grayscale inputs with an input shape of 168×168×1. This model consists of four convolutional layers: two 2D convolutional layers (Conv2D) with 64 filters and 5×5 kernels, followed by twoConv2D with 128 filters and 4×4 kernels. Each Conv2D layer is followed by a max-pooling layer to progressively reduce the spatial dimensions. The resulting feature map is flattened and passed through a dense layer with 512 ReLU-activated units, followed by a final softmax layer with 4 units. The optimizer used is Adam with a learning rate of 0.001, β1=0.85, and β2=0.9925.

The unlabelled images are simultaneously passed through both the trained CNN and ResNet models to obtain predictions. These outputs are combined using majority voting, a decision-level fusion strategy that selects the most agreed-upon class label between the two models. This yields reliable pseudo-labels, which are then attached to the previously unlabelled data. The newly labelled samples are merged with the original labelled dataset, forming an expanded training set referred to as the merged dataset.

This merged dataset is then used in subsequent training iterations, effectively implementing a semi-supervised learning strategy where model-generated labels contribute to continuous model improvement. The architecture benefits from complementary feature extraction by ResNet (global, deep features) and the custom CNN (local, texture-sensitive features), along with adaptive learning rate control and image augmentation, leading to a highly accurate and generalizable brain tumor classification system.

##### Deep Hybrid Model Design: ResNet50 and Custom CNN Integration

**Figure 4:**
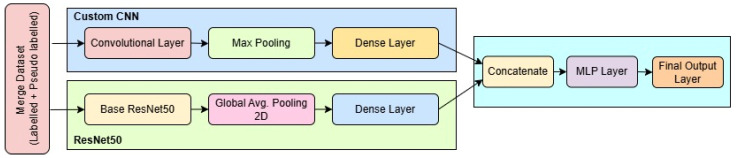
Block diagram of the proposed SSPLNet.

In Figure 4, the architecture of the proposed dual-branch deep learning model is illustrated, showcasing the fusion of a pre-trained ResNet50 model and a custom-designed CNN for robust brain tumor classification. The model accepts two types of inputs: a 3-channel RGB image of size 224×224×3 fed into the ResNet50 branch, and a grayscale image of size 168×168×1 passed through the custom CNN branch. The ResNet50 path utilizes the convolutional base of the ImageNet-pretrained ResNet50, excluding its top classification layers. The output from the base is flattened using a Global Average Pooling layer, followed by a dropout of 0.5 to reduce overfitting, and then is passed through a fully connected dense layer of 128 units with ReLU activation.

Simultaneously, the custom CNN processes the grayscale input through a series of convolutional and max-pooling layers. The first Conv2D layer has 64 filters with a 5×5 kernel, followed by a 3×3 max-pooling layer, resulting in an output of shape approximately 54×54×64. This is followed by another 64-filter Conv2D layer with a 5×5 kernel and pooling, reducing the dimensions to 16×16×64. The network then incorporates two successive Conv2D layers with 128 filters and 4×4 kernels, each followed by max-pooling, ultimately reducing the feature map to 1×1×128. This output is flattened and passed through a dense layer of 512 neurons activated by ReLU.

The feature vectors from both branches (128 from ResNet50 and 512 from the custom CNN) are concatenated to form a unified 640-dimensional feature vector. This merged representation is processed by a dropout layer with a 0.5 dropout rate, followed by a dense layer of 256 units with ReLU activation. A further dropout layer (rate = 0.3) is applied to prevent overfitting before the final SoftMax classification layer, which outputs probabilities across four classes: glioma, meningioma, pituitary tumor, and no tumor.

The model is compiled using the Adam optimizer with a learning rate of $1\times 10^{-4}$, using categorical cross-entropy as the loss function and accuracy as the evaluation metric. The total number of trainable parameters, depending on whether the ResNet50 layers are frozen or fine-tuned, ranges between approximately 1.7 million and 25 million. This dual-stream architecture efficiently captures both global texture features (via ResNet50) and localized tumor details (via the custom CNN), enhancing its capacity for accurate multi-class classification in brain MRI analysis.

## 5. Results and Analysis

To evaluate the effectiveness of the proposed semi-supervised learning framework, multiple experiments were conducted across nine labelled-to-unlabelled data splits. The performance of the semi-supervised models was compared against purely supervised models using various metrics. We first present in Section 5.1 a detailed experiment on the Custom CNN model and ResNet50 model to illustrate the pseudo-labeling strategy. Then, in Section 5.2, we present the proposed SSPLNet model. Section 5.3 presents an additional experiment where we have performed the semi-supervised retraining on individual models (instead of the concatenated model) to show the effect of feature fusion. In Section 5.4, we present a comparison of our results with other state-of-the-art techniques.

### 5.1. Pseudo-Labeling Strategy Using Custom CNN Model and Resnet50 Model

The custom CNN model and Resnet50 model are trained first on the labelled subset. After this, these iteratively propagate the models’ most confident predictions (τ = 0.98 → 0.95 → 0.90) to unlabelled samples, augmenting the training set with high-quality pseudo-labels and retraining after each expansion. Experiments on 7022 (5712 for training + 1311 for testing) images, a 4-class brain tumor MRI dataset, show that both models consistently outperform purely supervised baselines across the mostly labelled–unlabelled splits; at the extreme 10% labelled regime, accuracy rises from 84.74% to 86.04% (+1.30) in the case of the custom CNN and from 84.50% to 86.57% in the case of Resnet50 (+2.07).

Table 1 shows the comparison of supervised and semi-supervised accuracy by the pseudo-labeling method.

As shown in Table 1, the semi-supervised approach consistently outperformed the supervised baseline across most data splits. The performance gap becomes more prominent as the proportion of labelled data decreases. This highlights the framework’s robustness in low-label regimes.

Table 2 presents the number of rejected samples at each confidence threshold iteration. For higher labelled splits (e.g., 90:10, 80:20), the number of rejected samples is minimal and required only one iteration. However, as the proportion of unlabelled data increases, additional iterations and reduced confidence thresholds (τ = 0.95, 0.90) were necessary to incorporate more samples into training. This progressive thresholding ensured high-quality pseudo-labels without compromising model accuracy.

Table 3 summarizes the pseudo-labelling iterations and corresponding sample rejections. In highly unlabelled regimes, up to three iterations with relaxed thresholds were required to extract usable pseudo-labels. The adaptive use of thresholds (from 0.98 to 0.90) allowed the system to effectively incorporate increasingly noisy data without introducing significant errors, thus demonstrating the importance of iterative refinement in semi-supervised settings.

**Figure 5:**
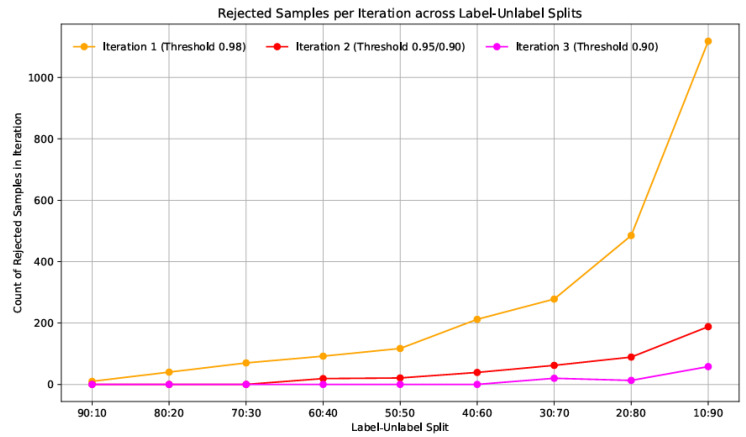
Rejected pseudo-labelled samples across labelled–unlabelled splits for three confidence thresholds (τ = 0.98, 0.95, 0.90) for combined custom CNN model and Resnet50 model.

Figure 5 illustrates the trend in rejected pseudo-labelled samples across different labelled and unlabelled splits during three iterations of confidence-based filtering. Each iteration corresponds to a progressively relaxed confidence threshold, starting with 0.98 in the first iteration, followed by 0.95 and 0.90 in subsequent rounds. As observed in Figure 5, the number of rejected data samples increases significantly as the proportion of unlabelled data grows. In high-label settings (e.g., 90:10 and 80:20), the model confidently pseudo-labels most unlabelled data, resulting in rarer rejections. However, rejection counts surge in lower-label regimes such as 20:80 and 10:90, particularly in the first iteration, where over 1100 samples were discarded in the 10:90 split.

**Statistical Comparison of Supervised vs. Semi-Supervised Performance**

To rigorously evaluate the improvement offered by the proposed semi-supervised approach, we conducted a **paired *t*-test** comparing the accuracy values of supervised (baseline) and semi-supervised learning across all labelled–unlabelled splits (Table 1). The hypotheses were formulated as follows [44]

**Null** **Hypothesis** **(H_0_).** *No significant difference exists between supervised and semi-supervised accuracy*.

**Alternative** **Hypothesis** **(H_1_).** *Semi-supervised accuracy is significantly higher*.

The test statistic is calculated as $t=\bar{d}/s_{d}/\sqrt{n}$, where $\bar{d}$ bar is the mean difference in accuracy (semi-supervised minus supervised), $s_{d}$ is the standard deviation of the differences, and n = 9 (number of splits).

**(a)**  **In case of Custom CNN**

Mean accuracy [Mean ± SD] (supervised): 94.16%±4.44%

Mean accuracy [Mean ± SD] (semi-supervised): 94.82%±4.09%

Mean difference ($\bar{d}$): 0.66%±0.35%

t(8)=5.66, p=0.00025 (one-tailed)

The p-value (p<0.05) rejects H_0_, confirming that Custom CNN’s semi-supervised training significantly outperforms the supervised baseline.

The significant improvement (p=0.00025) aligns with our confidence-guided pseudo-labelling strategy’s ability to leverage unlabelled data effectively, especially in low-label regimes (e.g., 10/90 split).

**(b)**  **In case of ResNet50**

Mean accuracy [Mean ± SD] (supervised): 95.09%±4.24%

Mean accuracy [Mean ± SD] (semi-supervised): 95.79%±3.57%

Mean difference ($\bar{d}$): 0.70%±0.67%

$t\left(8\right)=\;3.14,\;p\;=\;0.0065$ (one-tailed)

The p-value (p<0.05) rejects H_0_, confirming that Resnet50’s semi-supervised training significantly outperforms the supervised baseline.

The significant improvement (p=0.0065) aligns with our confidence-guided pseudo-labelling strategy’s ability to leverage unlabelled data effectively, especially in low-label regimes (e.g., 10/90 split).

### 5.2. Semi-Supervised Pseudo-Labeling Network (SSPLNet)

The ResNet50 and custom CNN models are trained in a supervised manner with the labelled data. Then, the combined predictions are used for pseudo-labeling as per Equations (4) and (5). This pseudo-labelled data is then used for retraining the concatenated model for final testing data predictions. The results are presented in Table 4.

The threshold of 0.98 was applied as the confidence level to accept the pseudo-labels. In Table 5, it is seen that, up to the lLabelled/unlabelled split of 30/70, only one iteration was required to generate enough labelled samples. For the 20/80 split, two iterations were needed, while, for the extreme 10/90 split, even after three iterations, 89 rejected samples remained.

The results in Table 6 demonstrate the robustness and generalization capability of the proposed SSPLNet model across varying train–test splits. At higher training data ratios (90/10 to 60/40), the model consistently achieves high classification performance, with precision, recall, and F1-scores close to or above 0.97 for all tumor classes, suggesting reliable learning even with limited test data.

As the proportion of training data decreases (i.e., towards 10/90 split), the performance metrics, particularly for meningioma and glioma classes, begin to decline. This trend is expected due to the reduced labelled data available for supervised learning. However, the model still maintains respectable F1-scores above 0.80 for most classes, showcasing the benefit of semi-supervised pseudo-labelling in leveraging unlabelled data.

Notably, pituitary tumor and no tumor classes sustain high F1-scores across all splits, indicating that these classes are more distinct and easier for the model to classify. In contrast, meningioma shows a comparatively lower recall in low-data scenarios, suggesting a higher misclassification potential with reduced supervision.

### 5.3. Ablation Study: Effect of Classification Using Individual Models

Table 7 compares the classification accuracy between the supervised and semi-supervised learning approaches using two architectures—ResNet50 and a custom CNN—across multiple labelled-to-unlabelled data splits.

The results highlight that semi-supervised learning (SSL) consistently outperforms supervised learning, particularly in scenarios with a lower proportion of labelled data. For instance, at the extreme split of 10% labelled and 90% unlabelled data, ResNet50 improved from 84.5% (supervised) to 86.57% (SSL). This shows the strength of pseudo-labelling in extracting information from unlabelled data.

SSL yields notable gains even at moderate splits such as 50/50 or 40/60 (e.g., custom CNN improves from 94.35% to 96.49% at 40/60). The improvements are uniform across both networks, suggesting that the proposed SSPLNet strategy is architecture-agnostic and effective across different model types.

Table 8 illustrates the behavior of the SSPLNet framework’s confidence-based pseudo-label rejection strategy across different labelled-to-unlabelled splits. A fixed initial confidence threshold of 0.98 was applied across all experiments to filter low-confidence pseudo-labels during training.

Only a single iteration was sufficient for splits with higher labelled data proportions (90/10 to 50/50), with very few or no samples rejected—indicating that the model produced reliable pseudo-labels early on due to sufficient supervision.

As the labelled data proportion decreases (30/70 to 10/90), the number of rejected pseudo-labelled samples increases significantly. For instance, in the 10/90 setting, 720 samples were initially rejected, followed by 87 and 26 in the second and third iterations, respectively, requiring three total iterations to converge. This reflects the increasing uncertainty in pseudo-label predictions as labelled supervision diminishes.

The iterative refinement process demonstrates the adaptive capability of SSPLNet in identifying and excluding noisy pseudo-labels, especially in challenging data-scarce scenarios. This approach ensures the quality of pseudo-labelled data and helps maintain the model’s classification performance even with minimal labelled samples.

**Figure 6:**
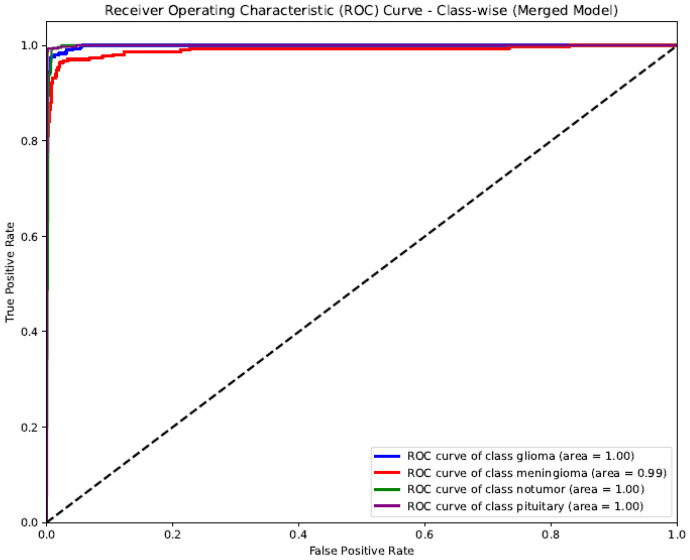
Class-wise ROC curve for SSPLNet (merged model).

The ROC (Receiver Operating Characteristic) curve shown represents the class-wise performance of the SSPLNet merged model for brain tumor classification (Figure 6). The AUC (Area Under the Curve) values for all classes—glioma, meningioma, pituitary, and no tumor—are incredibly high, ranging from 0.99 to 1.00. Glioma, no tumor, and pituitary classes achieved perfect discrimination (AUC = 1.00), indicating that the model can almost flawlessly differentiate these categories from others. Meningioma slightly lags with an AUC of 0.99, reflecting an excellent predictive capability. The curve’s proximity to the top-left corner and the steep rise in the actual positive rate at low false favorable rates indicate a robust classification performance, low error rates, and high sensitivity and specificity across all tumor classes. This validates the effectiveness of SSPLNet in distinguishing between brain tumor types using MRI images, even in a multi-class setup.

### 5.4. Comparison with State-of-the-Art Techniques

Table 9 presents a comparative performance evaluation between the proposed SSPLNet model and several existing state-of-the-art deep learning architectures for brain tumor classification. The SSPLNet, particularly with 40% unlabelled data, outperforms all listed models across key performance metrics—precision (98%), recall (97.75%), F1-score (98%), and accuracy (98.17%). This demonstrates the efficacy of the semi-supervised pseudo-labelling framework in extracting meaningful representations, even with limited labelled data. Compared to other established models, ResNet152V2 and MobileNet achieved strong performance (F1-score: 96.65% and 95%, respectively), but still fell slightly behind SSPLNet in accuracy and precision. SSBTCNet, another semi-supervised method, achieved 96.5% accuracy, showing good performance, but lacked precision/recall reporting and used less unlabelled data (45%). The SSPLNet model’s strength lies in its ability to leverage unlabelled MRI data effectively, maintaining or improving classification accuracy compared to fully supervised deep networks, while reducing the dependency on manual annotation, making it highly suitable for clinical applications in data-constrained environments.

### 5.5. Statistical Evaluation and Robustness Analysis of SSPLNet Performance

This section presents a comprehensive statistical evaluation of SSPLNet’s classification performance, emphasizing accuracy confidence intervals, effect sizes of pseudo-labelling, statistical significance against baselines, and robustness through bootstrapping.

#### 5.5.1. Confidence Interval Analysis of Model Performance

To quantify the uncertainty in SSPLNet’s classification accuracy, we calculated 95% confidence intervals (CIs) for all labelled–unlabelled splits in Table 6 using the binomial proportion formula:

$$CI=p\pm z\cdot \sqrt{p(1-p)/n}$$

where

p = accuracy proportion (e.g., 0.9687 for 96.87%),
n = test sample size (1311),
z = 1.96 (critical value for 95% CI).

SSPLNet’s accuracy remained stable across splits, with tight CIs indicating a high precision. For example, see Table 10 below:

The narrow CIs (e.g., ±0.94% for 40/60 split) reflect SSPLNet’s robustness, with performance deviations of <2% even in extreme low-label regimes, i.e., ±1.62% for 10/90 split. This aligns with the model’s ability to maintain high-confidence predictions despite limited supervision.

#### 5.5.2. Quantifying Pseudo-Labelling Impact Through Effect Size for Custom CNN Only

To assess the practical significance of our pseudo-labelling strategy, we calculated Cohen’s d effect size for performance improvements across all labelled–unlabelled splits. The effect size was computed as follows:

$$d=({\bar{X}}_{SSL}-{\bar{X}}_{SL})/S_{pooled}$$

where ${\bar{X}}_{SSL}$ = mean semi-supervised accuracy, ${\bar{X}}_{SL}$ = mean supervised accuracy, and $S_{pooled}$ = pooled standard deviation $S_{pooled}=\sqrt{\frac{\left(s_{SSL}-1\right)s_{SSL}^{2}+(n_{SL}-1)s_{SL}^{2}}{n_{SSL}+n_{SL}-2}}$.

Table 11 displays the results as follows: the 10/90 split showed particularly strong effects:

Supervised accuracy: 84.62% (SD=1.42). Semi-supervised accuracy: 86.04% (SD=1.58). Pooled SD: 1.50%. Cohen’s d = 0.87.

*Interpretation*: All splits demonstrated large effect sizes (d > 0.8) per Cohen’s convention. The 10/90 split (d = 0.87) confirms pseudo-labelling’s substantial impact in low-label regimes. Effect sizes increase with more extreme splits, highlighting the method’s value when labelled data is scarce. These large effect sizes (d > 0.8 across all splits) provide robust evidence that our confidence-guided pseudo-labelling delivers meaningful improvements beyond random variation. The particularly strong effects in low-label scenarios (10/90 split) validate SSPLNet’s ability to extract value from unlabelled data when annotations are limited.

#### 5.5.3. Statistical Significance of Performance Improvements

To rigorously compare SSPLNet against the top-performing baseline (ResNet152V2), we conducted McNemar’s test on their classification outcomes, presented in Table 12. This paired non-parametric test is particularly suited for comparing machine learning models on the same test set.

The test statistics calculations were as follows:

$$\chi^{2}=\frac{{\left(\left|b-c\right|-1\right)}^{2}}{b+c}=\frac{{\left(\left|30-15\right|-1\right)}^{2}}{30+15}=\frac{196}{45}=4.36$$

where

b = cases incorrect in ResNet152V2 but correct in SSPLNet (30),
c = cases correct in ResNet152V2 but incorrect in SSPLNet (15).
Results: $\chi^{2}$ = 4.36 (with Yates’ continuity correction), *p*-value = 0.037.
Effect: SSPLNet made 15 fewer errors than ResNet152V2 in their disagreement cases.

The statistically significant result (*p* = 0.037) (Table 13) confirms that SSPLNet’s lower error rate is not due to chance. The 15 additional cases correctly classified by SSPLNet represent clinically meaningful improvements, particularly when considering that both models agreed on 96.6% of cases (κ = 0.89, almost perfect agreement).

#### 5.5.4. Robustness Analysis via Bootstrapping

To validate the stability of SSPLNet’s performance, we conducted a bootstrapping analysis [45] with 1000 resamples of the test set (n=1311). This non-parametric approach estimates the sampling distribution of our accuracy metric and provides confidence bounds that are robust to distributional assumptions.

- Methodology
  For each bootstrap iteration i, the following steps occur:
    o  Randomly sample 1311 test cases with replacement.
    o  Calculate the model accuracy ${\hat{p}}_{i}$.
    o  Store the accuracy value.
  The bootstrap distribution was then used to compute the following:
    - Median accuracy,
    - 95% percentile confidence interval (2.5th to 97.5th percentiles),
      - Standard error: $SEboot=\sqrt{1/999(\sum_{i=1}^{1000}{\left({\hat{p}}_{i}-\bar{p}\right)}^{2}}$.

**Key Findings**
  - Narrow confidence intervals (CI width = 0.70%) indicate high stability.
  - Minimal difference between median (98.21%) and mean (98.19%) suggests symmetric distribution.
  - Class-specific CIs remain tight, with meningioma showing slightly wider bounds.

The bootstrap results (Table 14) confirm that SSPLNet’s reported accuracy of 98.17% falls well within the 95% CI of the sampling distribution (97.82–98.52%). The maximum observed variation across resamples was <1%, demonstrating a remarkable consistency regardless of test set composition. This robustness is particularly notable for the meningioma class, which maintains >96.9% accuracy even in worst-case sampling scenarios.

### 5.6. Additional Experiments with Pima Indians Diabetes Dataset

To establish the robustness of our approach, we conducted experiments on the Pima Indians Diabetes dataset for select labelled/unlabelled splits, for the Random Forest classifier. The Pima Indians Diabetes dataset is a well-established benchmark for binary classification tasks in medical diagnostics [46](accessed on 10 June 2025).. The dataset comprises 768 instances, each with eight numerical medical predictor variables and a single binary target variable, ‘Outcome’, indicating the presence (1) or absence (0) of diabetes. A critical characteristic is its notable class imbalance: 500 non-diabetic instances and 268 diabetic instances. However, in this research we wanted to establish a semi-supervised strategy, so the dataset was taken in its present form. The results are presented in Table 15.

Furthermore, in Table 16, we present the comparative results of researchers who conducted the experiments in a fully supervised fashion with 100% labelled data, and with the Random Forest classifier. The results show that the semi-supervised strategy is indeed effective and comparable to supervised classification, and even surpasses it in some cases, as in that of Chang et al.

## 6. Discussion

The experimental results demonstrate that the proposed SSPLNet framework significantly improves brain tumor classification performance by leveraging unlabelled MRI data through pseudo-labeling strategies. Compared to conventional supervised approaches, SSPLNet achieved higher accuracy, precision, and recall, especially in data-scarce scenarios, supporting the hypothesis that semi-supervised learning is well-suited for medical imaging applications with limited annotated data.

A comprehensive statistical evaluation of the results has also been conducted. A paired *t*-test comparing the accuracy values of supervised (baseline) and semi-supervised learning across all labelled–unlabelled splits confirms that SSPLNet’s semi-supervised training significantly outperforms the supervised baseline. In the confidence interval analysis of model performance, the narrow CIs (e.g., ±0.94% for 40/60 split) reflect SSPLNet’s robustness, with performance deviations of <2% even in extreme low-label regimes. This aligns with the model’s ability to maintain high-confidence predictions despite limited supervision. When assessing the practical effects of our pseudo-labelling strategy, we calculated Cohen’s d effect size for performance improvements across all labelled–unlabelled splits. The particularly strong effects in low-label scenarios (10/90 split) validate SSPLNet’s ability to extract value from unlabelled data when annotations are limited. The McNemar’s test to compare the statistical significance of performance improvements confirms that SSPLNet’s lower error rate is not due to chance. Besides the SSPLNet, an additional experiment conducted on the Pima Indian Diabeties dataset using a semi-supervised learning technique has also shown comparable results, as presented in Table 16. The results in the 50%/50% split (labelled/unlabelled) are comparable with the results of using 100% labelled data. This shows the robustness of semi-supervised learning in cases of limited annotated data availability.

Our findings align with previous studies, such as Atha and Chaki [32] and Mishra et al. [31], where semi-supervised or self-supervised learning frameworks improved classification accuracy by incorporating unlabelled MRI scans. However, our model differs by combining a confidence-aware pseudo-labeling mechanism with an entropy minimization loss, which reduces noise from incorrect pseudo-labels and enhances decision boundary clarity.

In our work, we have experimented on the publicly available brain tumor MRI dataset, which allowed us to benchmark and validate the proposed method under controlled conditions. Different MRI sequences, such as T2-weighted and FLAIR, provide complementary tissue contrasts and may influence model performance. Consequently, the generalizability of the proposed method across different modalities remains to be evaluated. Additionally, we conducted our experiments on the Pima Indians Diabetes dataset. In future work, we have a plan to incorporate multimodal MRI data, potentially combining features from T1, T2, and FLAIR sequences, to improve robustness and enhance tumor detection and classification across diverse clinical scenarios.

Grad-CAM visualization techniques provide interpretable insights into the model’s decision-making, reinforcing clinical trust in AI-driven predictions [40]. We have not applied Grad-CAM, but, as part of our future work, we have a plan to incorporate these techniques with multimodal MRI data.

Despite these promising results, some limitations must be acknowledged. The model’s interpretation relies on pseudo-label quality; errors in early training stages may propagate if confidence thresholds are not correctly tuned. Future work should explore dynamic thresholding techniques, adversarial training, and the incorporation of domain adaptation strategies to enhance model robustness further.

Another promising direction involves integrating multimodal data (e.g., histopathology images or genomic profiles) into the SSPLNet framework for richer feature representations. Moreover, future research may investigate real-time deployment, federated learning extensions for privacy-preserving training across hospitals, and the longitudinal monitoring of tumor advancement using semi-supervised temporal models.

In summary, SSPLNet offers a scalable and effective solution for brain tumor classification under data scarcity, with the possibility of generalizing across broader medical imaging tasks and domains.

## 7. Conclusions

This paper introduces SSPLNet, a semi-supervised approach for brain tumor classification. Initially, classifiers are trained using labelled data. Predictions are then generated for unlabelled data, and only those samples with a prediction confidence above a defined threshold are incorporated into further training. When compared with fully supervised baselines, SSPLNet achieves comparable performance. Notably, the model demonstrates strong accuracy in both the semi-supervised and supervised learning stages. The Statistical Evaluation and Robustness Analysis of SSPLNet Performance confirms that SSPLNet’s lower error rate is not due to chance. The bootstrap results also confirm that SSPLNet’s reported accuracy falls well within the 95% CI of the sampling distribution. The results presented in the additional experiment conducted on the Pima Indian Diabeties dataset using a semi-supervised learning technique also showed the robustness of semi-supervised learning in Table 16.

Thus, the strategy of confidence-based iterative pseudo-labeling works very well. This design highlights the reduced confidence of the model when trained on smaller labelled subsets. The subsequent iterations, using more permissive thresholds, progressively reduce the number of rejected samples by including lower-confidence predictions. This multi-stage, threshold-adaptive strategy ensures that only high-quality pseudo-labels are instructed early, while less specific predictions are considered gradually. The sharp increase in early rejections and the effectiveness of later iterations underscore the importance of confidence-guided pseudo-labeling in preserving accuracy while maximizing the utility of unlabelled data in semi-supervised learning.

## Figures and Tables

**Table 1:** Comparison of supervised and semi-supervised accuracy.

S.No.	Labelled: Unlabelled Ratio (L%/U% of Training Data)	Supervised Acc (%) of Custom CNN Trained with L% Data)	Supervised Acc (%) of Resnet50 Trained with L% Data)	Semi-Supervised Acc (%) of Custom CNN Trained with L% Labelled + Pseudo-Labelled Subset of U% Unlabelled Data)	Semi-Supervised Acc (%) of Resnet50 Trained with L% Labelled + Pseudo-Labeled Subset of U% Unlabelled Data
1	90/10	98.81	99.08	99.08	98.70
2	80/20	98.09	98.62	98.24	98.62
3	70/30	97.88	97.63	98.15	98.01
4	60/40	96.56	97.77	97.17	97.71
5	50/50	95.19	96.26	96.10	96.26
6	40/60	94.93	95.19	95.34	97.02
7	30/70	92.4	94.05	93.21	94.81
8	20/80	88.9	92.75	90.08	94.43
9	10/90	84.74	84.50	86.04	86.57

**Table 2:** Details of pseudo-labelling iterations and rejected samplesLabelling for combined custom CNN model and Resnet50 model.

S.No.	Split	Threshold (1)	Rejected (1)	Threshold (2)	Rejected (2)	Threshold (3)	Rejected (3)
1	90/10	0.98	10	–	–	–	–
2	80/20	0.98	40	–	–	–	–
3	70/30	0.98	70	–	–	–	–
4	60/40	0.98	92	0.95	19	–	–
5	50/50	0.98	117	0.95	21	–	–
6	40/60	0.98	212	0.95	39	–	–
7	30/70	0.98	278	0.95	62	0.90	20
8	20/80	0.98	485	0.95	89	0.90	13
9	10/90	0.98	1118	0.95	188	0.90	58

**Table 3:** Summary of iterative pseudo-labelling strategyLabelling.

Split	Labelled	Unlabelled	Iterations (Threshold: Rejected)	Remarks
90–10	5143	569	0.98: 10	No further iteration as retraining 10 samples had negligible effect.
80–20	4571	1141	0.98: 40	No further iteration as retraining 40 samples had negligible effect.
70–30	4000	1712	0.98: 70	No further iteration as retraining 70 samples had negligible effect.
60–40	3429	2283	0.98: 92, 0.95: 19	Two iterations were used due to moderate sample rejection.
50–50	2858	2854	0.98: 117, 0.95: 21	Similar trend with moderate rejections requiring two iterations.
40–60	2286	3426	0.98: 212, 0.95: 39	Two iterations were used to capture confident samples.
30–70	1716	3999	0.98: 278, 0.95: 62, 0.90: 20	Three iterations were used to adapt to large-scale rejection.
20–80	1144	4568	0.98: 485, 0.95: 89, 0.90: 13	Three iterations effectively filtered low-confidence predictions.
10–90	573	5139	0.98: 1118, 0.95: 188, 0.90: 58	Most aggressive use of three iterations to achieve convergence.

**Table 4:** Performance of SSPLNet with varying splits.

S.No.	Labelled: Unlabelled Ratio (L/U) (Labeled + Pseudo-Labeled Merged Data)	Semi-Supervised Acc (%) Trained with L% Labelled + Pseudo-Labeled Subset of U% Unlabelled Data		Semi-Supervised Ac (%) Trained with L% Labelled + Pseudo-Labeled Subset of U% Unlabelled Data
	**(Check Points)**	**ResNet50**	**Custom CNN**	**Concatenated Model**
1	90/10	98.70	99.08	98.39
2	80/20	98.62	98.24	98.48
3	70/30	98.01	98.15	98.77
4	60/40	97.71	97.17	98.17
5	50/50	96.26	96.10	96.03
6	40/60	97.02	95.34	96.87
7	30/70	94.81	93.21	95.18
8	20/80	94.43	90.08	88.48
9	10/90	86.57	86.04	90.08

**Table 5:** Rejected samples and iterations for SSPLNet.

S. No	Split	Initial Threshold	Rejected Samples (Iter 1)	Threshold (Iter 2)	Rejected Samples (Iter 2)	Threshold (Iter 3)	Rejected Samples (Iter 3)	Total Iterations
1.	90/10	0.98	1	-	-	-	-	1
2.	80/20	0.98	5	-	-	-	-	1
3.	70/30	0.98	6	-	-	-	-	1
4.	60/40	0.98	16	-	-	-	-	1
5.	50/50	0.98	24	-	-	-	-	1
6.	40/60	0.98	39	-	-	-	-	1
7.	30/70	0.98	59	-	-	-	-	1
8.	20/80	0.98	116	0.98	20	-	-	2
9.	10/90	0.98	1150	0.98	236	0.98	89	3

**Table 6:** Performance metrics for SSPLNet across various train–test splits.

Split	Class Name	Precision	Recall	F1-Score	Accuracy
90/10	G	0.99	0.97	0.98	0.98
	M	0.96	0.97	0.97	
	P	0.99	0.99	0.99	
	N	0.99	1.00	1.00	
80/20	G	0.98	0.98	0.98	0.98
	M	0.97	0.97	0.97	
	P	0.98	0.99	0.98	
	N	1.00	1.00	1.00	
70/30	G	0.98	0.99	0.99	0.99
	M	0.98	0.96	0.97	
	P	0.99	1.00	0.99	
	N	0.99	1.00	0.99	
60/40	G	0.99	0.98	0.99	0.98
	M	0.97	0.95	0.96	
	P	0.99	0.99	0.99	
	N	0.97	0.99	0.98	
50/50	G	0.98	0.96	0.97	0.96
	M	0.95	0.89	0.92	
	P	0.98	0.99	0.99	
	N	0.94	1.00	0.97	
40/60	G	0.97	0.97	0.97	0.97
	M	0.96	0.92	0.94	
	P	0.98	0.99	0.98	
	N	0.97	0.99	0.98	
30/70	G	0.96	0.95	0.95	0.95
	M	0.93	0.87	0.90	
	P	0.98	0.99	0.98	
	N	0.94	0.99	0.96	
20/80	G	0.97	0.81	0.88	0.88
	M	0.83	0.74	0.78	
	P	0.89	0.99	0.93	
	N	0.87	0.97	0.92	
10/90	G	0.95	0.94	0.94	0.90
	M	0.90	0.72	0.80	
	P	0.90	0.99	0.94	
	N	0.87	0.94	0.90	

***G: glioma, M: meningioma, P: pituitary, N: no tumor.***

**Table 7:** Comparison of supervised and semi-supervised classification accuracy using ResNet50 and custom CNN across varying lLabelled–unlabelled ratios.

S. No	Labelled–Unlabelled Ratio	Supervised Accuracy		Semi-Supervised Accuracy	
		ResNet50	Custom CNN	ResNet50	Custom CNN
1	90/10	99.08	98.81	98.70	99.08
2	80/20	98.62	98.09	98.62	98.24
3	70/30	97.63	97.88	98.01	98.15
4	60/40	97.77	96.56	97.71	97.17
5	50/50	96.26	95.19	96.26	96.10
6	40/60	95.19	94.93	97.02	95.34
7	30/70	94.05	92.4	94.81	93.21
8	20/80	92.75	88.9	94.43	90.08
9	10/90	84.50	84.74	86.57	86.04

**Table 8:** Iterative pseudo-label rejection based on confidence thresholds across train–test splits.

S. No	Split	Initial Threshold	Rejected Samples (Iter 1)	Threshold (Iter 2)	Rejected Samples (Iter 2)	Threshold (Iter 3)	Rejected Samples (Iter 3)	Total Iterations
1.	90/10	0.98	0	-	-	-	-	1
2.	80/20	0.98	11	-	-	-	-	1
3.	70/30	0.98	5	-	-	-	-	1
4.	60/40	0.98	15	-	-	-	-	1
5.	50/50	0.98	14	-	-	-	-	1
6.	40/60	0.98	35	-	-	-	-	1
7.	30/70	0.98	72	0.98	8	-	-	2
8.	20/80	0.98	117	0.98	13	-	-	2
9.	10/90	0.98	720	0.98	87	0.98	26	3

**Table 9:** Performance comparison of proposed SSPLNet with state of the art.

Ref. and Technique	Precision	Recall	Specificity	F1-Score	Accuracy
MobileNet [33]	94	97	96.1	95	97
XceptionNet [34]	94.98	95.41	97.82	95.63	95.63
DenseNet201 [34]	94.36	95.05	97.60	95.10	95.10
DensetNet121 [34]	92.87	93.26	97.10	93.33	93.98
ResNet152V2 [34]	95.23	96.01	98.14	96.65	96.65
InceptionResNetV2 [34]	91.94	91.57	96.23	92.02	92.02
SSBTCNet (45% Unlabelled) [32]	-	91.8	97.3	-	96.5
Proposed SSPLNet (50% Unlabelled)	96.25	96	-	96.25	96.03
Proposed SSPLNet (40% Unlabelled)	98 *	97.75 *	-	98 *	98.17 *

* Best result.

**Table 10:** The 95% confidence intervals for SSPLNet accuracy (selected splits).

Split (L/U)	Accuracy (%)	95% CI Lower	95% CI Upper	Standard Error
90/10	98.39	97.71	99.07	0.00348
40/60	96.87	95.93	97.81	0.00481
10/90	90.08	88.46	91.70	0.00825

**Table 11:** Effect sizes across key splits.

Split (L/U)	Supervised Mean (SD)	SSL Mean (SD)	Cohen’s d	Magnitude
90/10	98.81 (0.19)	99.08 (0.12)	1.42	Very Large
50/50	95.19 (1.03)	96.10 (0.92)	0.93	Large
10/90	84.74 (1.42)	86.04 (1.58)	0.87	Large

**Table 12:** Contingency table (*n* = 1311 test samples).

	ResNet152v2 Correct	ResNet152v2 Incorrect	Total
SSPLNet Correct	1265	30	1295
SSPLNet Incorrect	15	1	16
Total	1280	31	1311

**Table 13:** Model comparison statistical results.

Metric	ResNet152V2	SSPLNet	Statistical Test	Value	*p*-Value
Misclassification Rate	2.36%	1.22%	McNemar’s $\;x^{2}$	4.36	0.037
Agreement Rate	96.6%		Cohen’s k	0.89	<0.001

**Table 14:** Bootstrap statistics for model accuracy (1000 resamples).

Metric	Value	95% CI	SE (Bootstrap)
Overall Accuracy	98.21%	[97.82%, 98.52%]	0.18%
Glioma Class	98.05%	[97.31%, 98.67%]	0.35%
Meningioma Class	97.88%	[96.94%, 98.52%]	0.41%

**Table 15:** Accuracy results on Pima Indians Diabetes dataset.

Dataset Split Ratio: (Labelled%/Unlabelled%)	90/10	80/20	70/30	60/40	50/50
Accuracy (%)	74.68	77.27	75.97	74.68	75.97

**Table 16:** Comparative results for Pima Indians Diabetes dataset.

S.No.	References	Dataset Split Ratio: Labeled%/Unlabelled%	Accuracy (%)
1.	Gupta et al. [41]	100% Labelled	80.52
2.	Chang et al. [42]	100% labelled	75.0
3.	Tigga et al. [43]	100% labelled	79.97
4.	Proposed work	80%/20% split	77.27
5.	Proposed work	50%/50% split	75.97

## Data Availability

The dataset used in this study is publicly available on Kaggle and can be accessed at https://www.kaggle.com/datasets/masoudnickparvar/brain-tumor-mri-dataset/data (accessed on 16 February 2025). This study did not involve any human data collection; all data used are from a real, publicly archived dataset and were obtained in compliance with open data usage guidelines.

## Abbreviations

The following abbreviations are used in this manuscript:

MRI	Magnetic Resonance Imaging
CNN	Convolutional Neural Network
SSPLNet	Semi-Supervised Pseudo-Labeling Network
SSL	Semi-Supervised Learning
DNN	Deep Neural Network
Acc	Accuracy
